# Dialysis versus conservative kidney management in older adults: why one size does not fit all

**DOI:** 10.1007/s11255-026-05009-3

**Published:** 2026-01-15

**Authors:** Dragos Scripcariu, Andreea Covic, Loredana-Mariana Agavriloaei, Bogdan Dumitru Agavriloaei, Teodora Joghiu, Mihai Onofriescu, Alexandru Burlacu, Luminita Voroneanu, Mehmet Kanbay, Adrian Covic

**Affiliations:** 1 Department of Medicine, Division of Nephrology, Grigore T. Popa University of Medicine and Pharmacy Iasi, Iasi, Romania; 2 Nephrology Department, Clinical Hospital “Dr. C.I. Parhon”, Iasi, Romania; 3 Department of Medicine, Division of Nephrology, Koc University School of Medicine, Istanbul, Turkey; 4 Regional Institute of Oncology, Iasi, Romania; 5 Institute of Cardiovascular Diseases “Prof. Dr. George I.M. Georgescu”, Iași, Romania; 6 Department of Neurosurgery, Grigore T. Popa University of Medicine and Pharmacy Iasi, Iasi, Romania

**Keywords:** Conservative kidney management (CKM), Dialysis, Older adults, Frailty, Quality of life (HRQoL), Prognostic tools/shared decision-making

## Abstract

**Background:**

As the global population ages, an increasing number of older adults progress to end-stage kidney disease (ESKD). In this population, frailty, multimorbidity, and functional decline often limit the survival benefit of dialysis, challenging the conventional approach to renal replacement therapy.

**Purpose:**

To summarize current evidence comparing dialysis with conservative kidney management (CKM) in older adults with advanced chronic kidney disease (CKD), focusing on survival, quality of life, hospitalization, and prognostic tools.

**Methods:**

A narrative synthesis was conducted based on observational, cohort, and systematic review studies including adults aged ≥ 70 years with stage 4–5 CKD. The literature search was performed exclusively in the PubMed database, which represents a methodological limitation of this review. Search terms included: end-stage renal disease, chronic kidney disease, kidney failure, dialysis, conservative management, frailty, geriatric patients, and elderly patients. Outcomes were grouped into four domains: survival, quality of life, healthcare utilization, and prognostic models.

**Results:**

Across studies, dialysis prolonged survival mainly in younger and less comorbid patients, but this advantage diminished with increasing frailty and multimorbidity. CKM provided comparable or superior health-related quality of life (HRQoL) and was associated with fewer hospitalizations. Patients managed conservatively were more likely to die at home, reflecting closer alignment with end-of-life preferences. Prognosis was primarily determined by patient-level factors—age, frailty, and eGFR decline—rather than by treatment modality. CKM-specific prognostic models remain limited.

**Conclusion:**

In older adults with advanced CKD, survival gains from dialysis are modest and frequently offset by higher treatment burden. CKM offers a patient-centered alternative focused on quality of life, comfort, and goal-concordant care. The development of validated CKM-specific prognostic tools is essential to support individualized, evidence-informed decision-making.

## Introduction

Kidney failure (KF) represents an increasingly important global public health burden, driven largely by population aging and the rising prevalence of hypertension and diabetes mellitus. Epidemiological analyses, including the Global Burden of Disease Study, indicate that chronic kidney disease (CKD) is now among the leading global causes of mortality [1]. Consequently, a growing proportion of older adults progress to kidney failure and initiate kidney replacement therapy (KRT), despite high levels of frailty, multimorbidity, and functional decline, factors that cast doubt on the net benefit of dialysis in this subgroup [2, 3].

Although dialysis remains the conventional treatment for KF, it entails substantial burdens, procedural invasiveness, prolonged recovery, frequent hospitalizations, and reduced health-related quality of life, especially among elderly, dependent, and comorbid patients [2, 4, 5]. In many cases, survival gains may be modest, while detriments to functional independence are considerable [2, 3]. As a result, non-dialytic strategies such as conservative kidney management (CKM), also termed conservative care (CC) or maximum conservative management (MCM), have emerged as viable alternatives focused on symptom control, quality of life, and multidisciplinary support rather than life prolongation [3, 4].

Decision-making between dialysis and CKM necessarily extends beyond survival to include health-related quality of life (HRQoL), symptom burden, hospitalization frequency, treatment burden, and alignment with patient and family preferences, together with considerations of resource utilization [2, 3, 5, 6]. The increasing prevalence of frailty, defined as reduced physiological reserve and increased vulnerability, commonly characterized by weakness, exhaustion, slowed performance, low activity, and unintentional weight loss, further underscores the need for multidimensional assessment to guide modality choice and prognostication [6–8]. Pre-frailty, defined by 1–2 Fried criteria, identifies individuals at heightened risk of decline, and is particularly relevant in CKD management [9]. Our review encompasses older adults across the spectrum of vulnerability, not solely those who are frail.

Observational evidence suggests that in selected older, multi-morbid patients, the survival advantage of dialysis may diminish or disappear, whereas CKM may offer benefits in patient-centered outcomes such as hospital-free days and place of death [2, 3, 5]. Nonetheless, major gaps persist in prognostic tools: current models primarily estimate mortality for dialysis recipients, with few or no individualized models for conservative kidney management or for direct treatment-specific prognostic comparison at the point of decision-making [10].

This narrative review aims to synthesize current evidence comparing dialysis and conservative kidney management (CKM) in older adults with advanced CKD, focusing on survival, health-related quality of life, hospitalization rates, and available prognostic tools. Given the substantial heterogeneity among the included studies, a narrative rather than quantitative synthesis was undertaken. Accordingly, this review explores three interconnected aspects: first, the impact of treatment modalities on patient survival; second, the evaluation of patient-centered and surrogate outcomes, including health-related quality of life, symptom burden, and hospitalizations; and third, the identification of practical considerations to support informed and individualized clinical decision-making.

## Methods

The literature search was conducted exclusively in the PubMed database, which represents a methodological limitation of this review. Search terms included: advanced cancer, terminal cancer, end-stage cancer, terminal neoplastic disease, end-stage kidney disease (ESKD), chronic kidney disease, kidney failure, dialysis, conservative management, maxim conservative management, frailty, geriatric patients, and elderly patients. The search covered publications from database inception to August 2025.

Screening was performed independently by two reviewers (M.A. and Z.C.). In cases of uncertainty or disagreement about study inclusion, a third reviewer (A.B.) was consulted, and consensus was reached through discussion.

Studies were considered eligible if they investigated adult patients (≥ 18 years) with kidney failure, with particular focus on elderly or geriatric populations. Only studies that directly compared dialysis treatment with conservative kidney management (including maxim conservative management) were retained. The main outcomes of interest were survival, hospitalization rates, health-related quality of life, and the identification of prognostic indicators. Eligible study designs included observational, retrospective, and cohort studies, as well as systematic reviews, prognostic model studies, and large-scale database analyses. Case reports, editorials, and commentaries were excluded.

To ensure transparency, predefined inclusion and exclusion criteria guided the selection process.

Titles and abstracts were initially screened against these criteria. If eligibility was unclear at this stage, full texts were retrieved. Articles were subsequently excluded if, upon full-text review, they did not meet all requirements.

From 1,287 records identified in PubMed, 1,287 titles and abstracts were screened. Of these, 910 records were excluded for not meeting the predefined criteria. The remaining 377 full-text articles were assessed for eligibility. After full-text review, 366 articles were excluded, most commonly for the wrong population (no RF or age < 18 years), lack of a direct comparison between dialysis and conservative kidney management, ineligible study design (e.g., case reports, editorials, commentaries), language other than English or lack of full-text access. Eleven studies met all inclusion criteria and were retained in the qualitative synthesis: seven observational studies, one systematic review/meta-analysis, one survey/vignette study, one prognostic model development/validation study, and one retrospective nationwide database study.

Although restricting the literature search to PubMed represents a methodological limitation, its expected impact is minimal. PubMed provides access to the core peer-reviewed biomedical literature, including the primary journals publishing research on advanced CKD, dialysis, and conservative kidney management. In line with the objectives of this narrative review, to synthesize existing evidence rather than perform an exhaustive systematic search, the use of a single database was deemed appropriate for identifying studies most relevant to the clinical questions addressed.

## Results

**Fig. 1:**
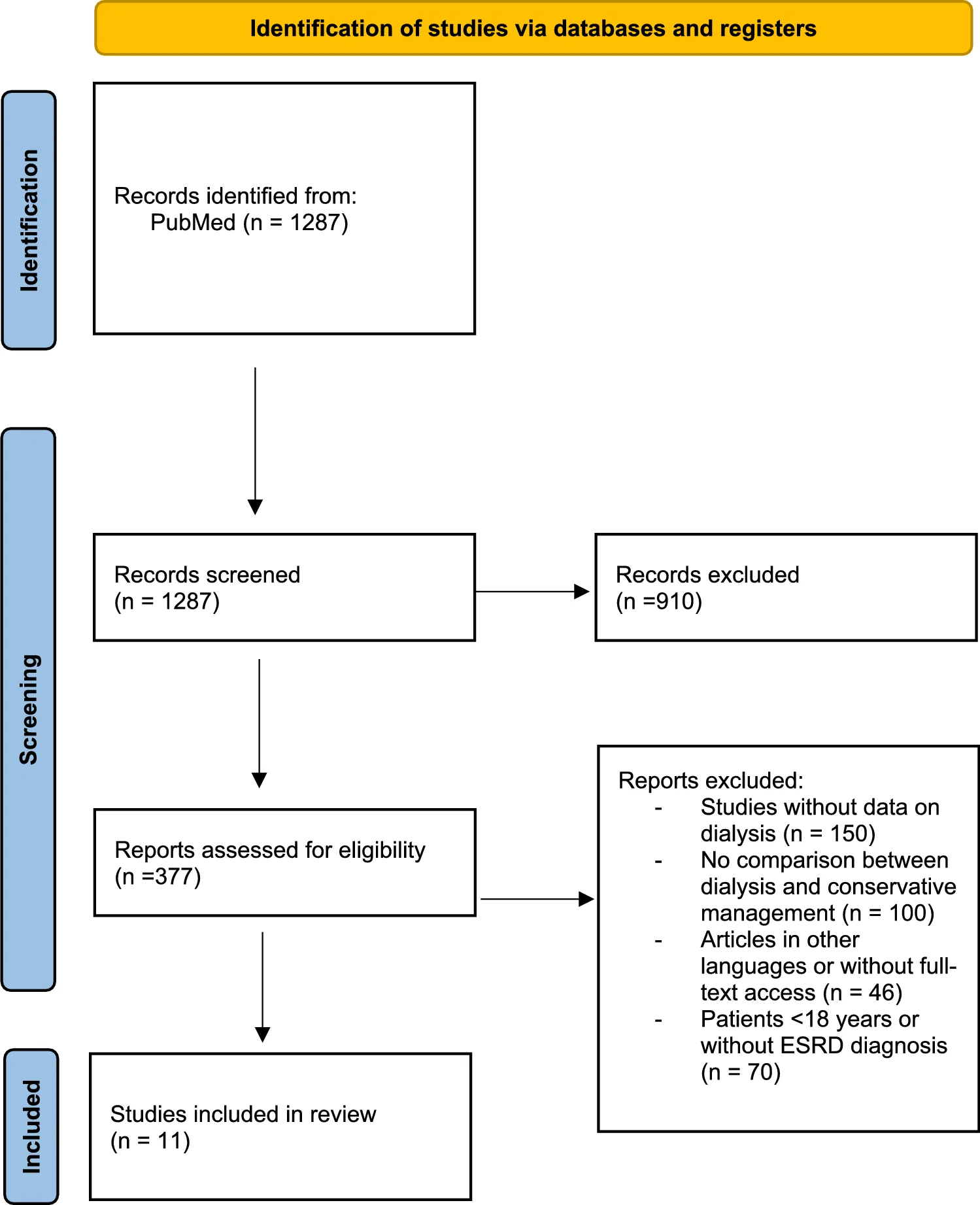
PRISMA flow diagram of study selection

The study selection process is illustrated in Fig. 1. A total of 1,287 records were identified through the PubMed database. After title and abstract screening, 377 full-text articles were assessed for eligibility, of which 11 studies met the inclusion criteria and were included in the qualitative synthesis.

The reviewed evidence demonstrates that outcomes under dialysis and conservative kidney management (CKM) diverge substantially according to patient frailty, comorbidity, and functional reserve. Across studies, dialysis confers a survival advantage mainly in younger, less comorbid individuals, whereas in frail older adults, CKM often provides comparable, or more favorable, patient-centered outcomes. A total of eleven studies evaluated dialysis versus CKM in older adults with kidney failure, approaching the question from complementary perspectives. Four studies directly compared hemodialysis with CKM in elderly patients [2, 4, 6, 7], while one study developed a prognostic tool to estimate 2-year mortality under both dialysis and conservative pathways [10]. One systematic review assessed health-related quality of life and symptom burden [3], and one survey study explored physician recommendations, revealing marked variability and noting that physicians were often more likely to choose dialysis for themselves than for their patients [5]. Two additional studies focused on dialysis withdrawal and end-of-life patterns [11, 12], and two examined factors influencing treatment choice beyond survival [1, 8]. Collectively, these findings indicate that frailty, age, and comorbidity critically shape both treatment selection and prognosis.

The key characteristics and main findings of the included studies are summarized in Table 1.

**Table 1:** Comparative summary of key studies comparing dialysis vs. conservative kidney management (CKM) in older adults

Ref	First author (year, country)	Design/domain	Population (N; age)	Comparison/focus	Follow-up	Primary outcomes	Key findings	Clinical implication
[1]	Álvarez-García (2023, Spain)	Observational—treatment choice and profiles	CKD stage 4–5; N = 211; older adults	In-center HD vs home-based KRT vs CKM; role of comorbidity/nutrition	Retrospective (multi-year)	Initial modality choice: nutritional and inflammatory markers	Higher comorbidity and lower albumin linked to in-center HD; better nutrition and lower CRP linked to home-based KRT; older/frailer patients more often chose CKM	Use CGA; optimize nutrition/inflammation when counseling
[2]	Chandna (2016, UK)	Observational—eGFR decline, modality, and survival	≥ 75 y; N = 250; progressive CKD (eGFR 10–15)	Dialysis vs CKM; impact of eGFR decline rate	≥ 3 years	Survival; rate of eGFR decline (time-dependent)	Dialysis yielded ~ 5-month adjusted survival advantage only in high comorbidity; rate of eGFR decline independently predicted mortality under CKM, not under dialysis	Trajectory of kidney function is prognostic for CKM; include decline rate in counseling
[3]	Verberne (2021, Netherlands)	Systematic review—HRQoL and symptoms	11 studies; N = 1,718; older KF	Dialysis vs CKM	To Oct 2019	HRQoL domains; symptoms	Physical health initially higher pre-dialysis but converges after start; mental health similar; kidney-disease-specific QoL often higher with CKM; dialysis start increases burden	CKM can achieve similar HRQoL; discuss treatment burden explicitly
[4]	Carson (2009, UK)	Observational—survival and utilization	≥ 70 y; single center	Dialysis vs CKM	NA	Survival; hospitalization; place of death	Dialysis extended median survival but increased hospitalizations; CKM associated with home/hospice death	Trade-off: longevity vs hospitalization and place of death
[5]	Finkelstein (2017, Indonesia)	Survey/vignette—physician decision-making	Nephrologists; vignette scenarios	Recommendations for dialysis vs CKM across profiles	Cross-sectional	Recommended modality; perceived survival	High variability; many recommend dialysis even in poor-prognosis cases; physicians more likely to choose dialysis for themselves than for patients	Need decision aids/prognostic tools to reduce variability
[6]	Lai (2020, Taiwan)	Retrospective—utilization in terminal cancer	Hospice decedents; *N* = 5,482 (HD 4,484; no HD 998)	Impact of HD during hospice	Claims-based	LOS; costs; death location	HD associated with longer hospice stay, higher costs, and in-hospice death; limited survival advantage	Prefer supportive CKM/withdrawal strategies in terminal cancer with KF
[7]	Murtagh (2007, UK)	Observational—survival by comorbidity	CKD5 ≥ 75 yr; *N* = 129	Dialysis vs conservative kidney management	Up to 2 years	1–2-yr survival; comorbidity strata	Dialysis improved survival overall (84% vs 68% at 1 yr), but advantage disappeared with high comorbidity, especially ischemic heart disease	Comorbidity (esp. IHD) should weigh heavily in counseling
[8]	Rodríguez‑Villarreal (2014, Spain)	Geriatric cohort—profiles, survival, frailty	≥ 75 yr; *N* = 56 (CKM 20; ITD 36)	CKM vs intention-to-dialysis (ITD)	Prospective	Survival; hospitalizations; frailty progression	Age and pre-frailty predicted CKM; no survival difference (log-rank p = 0.098); CKM had more hospitalizations and greater frailty progression	Functional reserve drives modality; monitor frailty trajectories
[9]	Ramspek (2021, Netherlands)	Prediction model—2-yr mortality	CKD 4/5 ≥ 70 y; N = 366 (126 CKM)	Separate models for dialysis vs CKM	2 years	2-year mortality; C-stat; calibration	Model (age, eGFR, CVD, malignancy) predicted 2-yr mortality for both pathways; C-stat 0.675–0.750; good calibration	Provide individualized risk estimates for SDM
[10]	Ko (2019, US)	Registry—withdrawal and end-of-life (≥ 80 y)	Incident HD ≥ 80 y; *N* = 17,296	Predictors/patterns of withdrawal	2007–2011	Withdrawal frequency; time to death; predictors	≈10% withdrew; withdrawal was 2nd–3rd cause of death; median 10 days from last HD to death; higher odds with dementia, catheter, malnutrition–inflammation; minorities less likely	Plan for possible withdrawal; address nutrition/access; discuss goals early
[11]	Kurella Tamura (2009, US)	Linked registry—functional trajectory	US nursing home residents starting dialysis; *N* = 3,702	Pre- vs post-dialysis function (MDS–ADL)	Up to 12 months	Function trajectory; survival	Sharp functional decline after initiation; by 12 months 58% died; only 13% maintained baseline function	Discuss functional trade-offs with frail elders before starting dialysis

ACKD, advanced chronic kidney disease; CKM, conservative kidney management; KRT, kidney replacement therapy; HD, hemodialysis; ITD, intention-to-dialysis; HRQoL, health-related quality of life; CGA, comprehensive geriatric assessment; ADL, activities of daily living; MDS-ADL, minimum data set-activities of daily living

### Patient characteristics, survival, and prognosis

Across the broader literature synthesis, conservative kidney management cohorts were older on average [CKM: 83 ± 4.43 vs. ITD (intention-to-dialysis): 78 ± 4.38], more likely to have multiple chronic conditions, and frequently functionally impaired, while dialysis cohorts tended to be younger, fitter, and less dependent at baseline. Reported comorbidities spanned cardiovascular disease (ischemic heart disease, heart failure, atrial fibrillation, peripheral vascular disease, stroke), diabetes, cerebrovascular disease/dementia, and depression, with several studies showing a higher overall comorbidity burden in CKM than in dialysis cohorts [3, 8]. Baseline characteristics and survival outcomes of the included cohorts are detailed in Table 1.

In a Spanish geriatric cohort, older age and pre-frailty independently predicted the selection of conservative kidney management, while cognitive impairment and dependence in activities of daily living were also more common. These findings highlight that functional reserve, rather than kidney function alone, often determines treatment choice in late life [8].

Nutritional and comorbidity profiles also shaped modality selection: patients who underwent in-center kidney replacement therapies had lower serum albumin (3.7 ± 0.6 vs. 4.0 ± 0.4 g/dL), reinforcing the observation that both biological vulnerability and functional decline influence decisions regarding dialysis initiation.

#### Survival

Four studies directly compared survival between hemodialysis and CKM. Collectively, they indicate that while dialysis can prolong life in selected patients, the benefit is modest and often vanishes with substantial comorbidity. In UK cohorts of patients aged ≥ 75 years, dialysis conferred higher 1- and 2-year survival than CKM, but this advantage was lost among those with high comorbidity, particularly ischemic heart disease [7].

Another analysis showed only a 5-month adjusted survival gain with dialysis in highly comorbid patients and identified the rate of eGFR decline as a stronger mortality predictor for CKM but not for dialysis [2].

Conversely, a Spanish geriatric cohort study found no significant survival difference between conservative kidney management (CKM) and dialysis (log-rank p = 0.098). However, patients managed conservatively experienced more frequent hospitalizations and greater progression to frailty during follow-up, while overall mortality rates were similar between the two groups [8].

A single-center UK cohort of patients ≥ 70 years likewise found longer median survival with dialysis but more hospitalizations, while CKM was associated with a higher chance of dying at home/hospice [4].

Overall, studies show a clear survival benefit of dialysis in patients who are younger and/or with lower comorbidity burden, but this benefit diminishes at older ages (≥ 75–80 years) and is lost in those with high comorbidity. For example, in cohorts over 75 years, dialysis conferred a median survival advantage (36 vs. 25 months), yet among patients with severe comorbidity, the difference was no longer significant (26 vs. 21 months), and in very frail geriatric series, no survival advantage was observed, although hospitalizations were more frequent in the conservative kidney management group. These nuances, advanced age, and comorbidity burden should be considered as central points in therapeutic decision-making at the bedside. The overall survival and hospitalization is presented in Table 2.

**Table 2:** Summary of survival and hospitalization presented in the studies

Study	Survival	Hospitalization
Kang et al., 2019	NA	HD group: • Mean hospice stay: 14.3 ± 12.3 days Non-HD group: • Mean hospice stay: 6.2 ± 6.9 days Difference: statistically significant (*p* < 0.001)
Álvarez-García et al., 2023	NA	NA
Chandna et al., 2016	• Survival was calculated from entry into CKD stage 5 (first eGFR 10–15 ml/min/1.73 m^2^) until death, transfer, or end of follow-up (minimum 3 years) Unadjusted survival • Dialysis pathway: median survival 38.2 months (95% CI 27.7–46.4) • Conservative kidney management (CKM): Median survival 23.1 months (95% CI 19.8–26.6) • Difference statistically significant (p < 0.001)	NA
Finkelstein et al., 2017	NA	NA
Verberne et al., 2021	NA	NA
Ramspek et al., 2020	Observed 2-year mortality (from follow-up data; Results—follow-up data) • Dialysis group: o 33% died within 2 years • Conservative care group: o 56% died within 2 years Median follow-up: • Dialysis: 37 months (IQR 17–58) • CC: 17 months (IQR 9–34)	NA
Kurella Tamura et al., 2009	Cumulative mortality after dialysis initiation, not median survival Mortality rates: • 24% at 3 months • 41% at 6 months • 51% at 9 months • 58% at 12 months	NA
Ko et al., 2019	NA	NA
Rodríguez-Villarreal et al., 2014	Survival calculated from treatment decision (intention to treat with dialysis vs conservative care) No statistically significant difference in survival • Log-rank *p* = 0.098 Mortality during follow-up: • Conservative care**:** 5/20 (25%) • Dialysis (ITD)**:** 6/36 (17%) • Difference not significant	Hospitalizations were more frequent in the conservative care group • CC: 16/20 (80%) • ITD: 17/36 (47%) • *p* = 0.017
Ramspek et al., 2021	Two-year mortality (primary study outcome) • Dialysis: 33% mortality at 2 years • Conservative care (CC): 56% mortality at 2 years	NA
Lai et al., 2020	Mortality increased significantly with higher MPI class: • MPI 0 (low risk): 0 deaths • MPI 1 (moderate risk): 22 deaths • MPI 2 (severe risk): 11/11 patients died	Differences between MPI classes • MPI 0: • o median of 0 hospitalizations/year (IQR 0–0) • MPI 1**:** o median of 1 hospitalization/year (IQR 1–2) • MPI 2**:** o median of 4 hospitalizations/year (IQR 3–4) • The differences were statistically significant**:** o *p* < 0.001 across all classes

Across included studies, survival outcomes were predominantly reported using Kaplan–Meier survival analysis, with median survival derived from survival curves rather than fixed-time survival rates. Direct numerical survival percentages were inconsistently reported.

#### Prognosis

Prognosis was driven chiefly by patient-level risk rather than modality. The rate of eGFR decline independently predicted mortality in conservative kidney management, but not in those starting dialysis, highlighting disease trajectory as a key prognostic signal in the non-dialysis pathway [2]. Frailty or pre-frailty, cognitive impairment, and ADL dependence not only steered decisions toward conservative kidney management but were also associated with a higher hospitalization burden [8].

A multivariable prediction tool further demonstrated that individualized 2-year mortality estimates differed for the same patient under dialysis versus CKM, based on age, eGFR, cardiovascular disease, and malignancy, with moderate discrimination (C-statistics ~ 0.68–0.75) [10]. Complementary to this, the Multidimensional Prognostic Index (MPI) showed strong associations with hospitalizations and mortality across both pathways. Overall, frailty, comorbidity burden, and renal function trajectory outweighed the dialysis-versus-CKM distinction in shaping outcomes [6], and although physical functioning may initially appear superior in patients preparing for dialysis, health-related quality-of-life trajectories tend to converge over time. Notably, kidney-disease-specific quality of life often favors CKM, reflecting reduced treatment burden and greater autonomy.

### Health-related quality (HRQoL) of life and symptoms

In patients with kidney failure (KF), the focus has increasingly shifted from merely prolonging life to enhancing its health-related quality. While dialysis may extend survival, it is frequently associated with physical discomfort, dietary restrictions, and frequent hospital visits, which can negatively impact daily functioning and social life, especially in older patients. Conservative kidney management has been proposed as an alternative in this category of patients. In a systematic review comprising 11 observational studies and a total of 1,718 patients, the HRQoL of individuals with CKD was evaluated, comparing those who chose conservative kidney management (CKM) with those who opted for dialysis. Physical health outcomes, assessed using validated instruments such as the 36-item Short Form (SF-36) and the 12-item Short Form (SF-12), were found to be lower in patients who selected CKM compared to those who had chosen dialysis but had not yet initiated treatment. These differences were evident in domains such as physical function, general health, and the physical component summary. However, once dialysis was initiated, physical health outcomes between the two groups became comparable, regardless of dialysis modality [3].

The symptom burden, as reflected in mental health outcomes, was found to be similar across both groups. This includes domains such as the mental component summary, vitality, social functioning, emotional role, and overall mental health. Over the long term, based on repeated assessments conducted over 12–36 months, mental health outcomes followed a similar trajectory in both groups, with no significant differences observed.

These findings suggest that while initial physical health may differ, long-term HRQoL trajectories in both physical and mental domains tend to align across treatment pathways. However, three studies that specifically evaluated kidney disease-related quality of life showed that patients who chose conservative kidney management reported better scores than those on dialysis, particularly in domains related to the impact of kidney disease on daily life and perceived burden of illness. Moreover, life satisfaction and general health status were found to decline after the initiation of dialysis, suggesting that starting dialysis may negatively affect certain aspects of well-being.

The questionnaires used in these studies for evaluation of health-related quality of life were SF-36, KDQOL-36, and KDQOL-SF. The SF-36 is a widely used generic health-related quality of life (HRQoL) questionnaire applicable across various medical conditions and populations. It consists of 36 items that assess eight key health domains (physical functioning, role physical, bodily pain, general health perceptions, vitality, social functioning, role emotional, mental health). The KDQOL is a disease-specific HRQoL instrument designed for patients with CKD, incorporating domains that specifically address the impact of kidney disease. There are two main versions: KDQOL-SF—combines SF-36 with kidney-specific items and KDQOL-36—a shorter version with 36 items, widely used in clinical settings.

### Use of healthcare services and hospitalization rates

Patterns of healthcare utilization further differentiate CKM and dialysis pathways, particularly in terms of hospitalization frequency and place of death.

Regarding hospitalizations, we identified three studies that investigated this outcome. Two of them reported a higher frequency of hospital admissions among patients undergoing dialysis, while one study found increased hospitalization rates in patients receiving conservative kidney management (CKM). Carson et al. conducted an observational study involving 202 patients with a mean age over 70 years. Of these, 79 received conservative kidney management (CKM), while 123 underwent kidney replacement therapy. The study found a higher rate of hospitalization among patients treated with dialysis (0.069 days per patient versus 0.043 days in the conservatively managed group). This hypothesis, suggesting more frequent hospitalizations among dialysis patients, was further supported by a smaller observational study conducted on 56 patients, which reported similar trends in hospitalization rates favoring conservative kidney management (CKM) [8].

Regarding mortality, patients in the conservative treatment group were 4.5 times more likely to die at home compared to those receiving dialysis. When balancing age and associated comorbidities, death at home may, in some cases, reflect a greater degree of psychological comfort for the patient, potentially translating into a better perceived health-related quality of life.

In a large-scale study conducted in Taiwan involving 5,482 cancer patients, 4,484 undergoing hemodialysis, and 998 managed conservatively, patients receiving dialysis had a higher likelihood of hospitalization, thus supporting findings from previous studies [12].

Overall, dialysis is consistently associated with a higher hospitalization burden, while CKM offers greater potential for end-of-life care congruent with patient preferences. Hospitalization patterns across studies are summarized in Table 1.

### Therapeutic decisions and predictive factors

The decision to pursue dialysis or conservative kidney management (CKM) appears to be influenced by multiple factors, ranging from patient frailty and social status to the presence of comorbid conditions.

Beyond patient-level characteristics, physician perception and prognostic uncertainty also shape treatment recommendations.

In a small study involving 56 patients from nephrology centers in Madrid, Spain, the choice of conservative kidney management (CKM) over hemodialysis was more likely among elderly, frail individuals who were dependent on assistance and exhibited cognitive impairment. The presence of cardiovascular comorbidities did not appear to influence the treatment decision. However, patients with diabetes mellitus were more frequently found in the dialysis group. Based on this study, we conclude that patients who are older, more dependent for ADL (activities of daily living), and have more cognitive deficits are more likely to opt for conservative kidney management (CKM) over dialysis [8].

Further analysis of an observational study conducted in a UK renal center (Lister Hospital, Stevenage, United Kingdom) highlighted the importance of kidney function decline, measured by time-dependent estimated glomerular filtration rate (eGFR td), in predicting survival among CKM patients. In patients with high comorbidity, dialysis was associated with a non-significant adjusted survival advantage of 5 months. However, among CKM patients, those at the 25th percentile of eGFR td had an adjusted survival of just 7 months, compared to 63 months for those at the 75th percentile. This suggests that the rate of kidney function decline is a critical determinant of both treatment choice and mortality risk in conservatively managed patients [2].

Given the trust patients often place in their physicians, medical advice frequently plays a significant role in shaping treatment decisions. Building on this premise, Eric A. Finkelstein et al. conducted an interesting study in which they surveyed physicians attending the 9th Asian Forum of the Chronic Kidney Disease Initiative conference. The study used vignettes that vary by age and comorbidity status, and asked physicians to recommend dialysis or conservative kidney management (CKM) for a hypothetical patient with that profile and to predict survival with both treatment options. The physicians were predominantly mid-career clinicians (mean age 45, 60% male), representing a mix of internists, general practitioners, and nephrologists, with about half affiliated with public hospitals and an average of 8 years’ ESRD experience.

Physicians generally believe that dialysis offers greater survival benefits than conservative kidney management (CKM), regardless of patient profile. However, a notable proportion of physicians (up to 50% in some scenarios) believed CKM could offer better survival than dialysis, especially in cases involving advanced cancer. Surprisingly, 25% of physicians still recommended dialysis for patients with advanced cancer. Even in scenarios where dialysis is expected to be most beneficial (e.g., younger patients with diabetes), only 62% of physicians recommended it. As age and comorbidities increase, the percentage recommending dialysis drops significantly (e.g., 34% for an 85-year-old with diabetes and CHF). Taken together, older age (85 years), advanced cancer, and low socioeconomic status decreased the likelihood of recommending dialysis [5].

These findings underline how personal beliefs and risk interpretation among clinicians contribute to treatment variability, underscoring the need for standardized CKM prognostic tools.

Physicians who were more optimistic about dialysis outcomes were more likely to recommend it, even in poor-prognosis cases. This highlights the role of personal beliefs and biases in clinical decision-making. When considering treatment for themselves, physicians were less likely to choose dialysis if they had advanced cancer, but more likely if they were 75 years old or middle-class. Thus, when physicians are asked what treatment they would choose for their patients, it becomes evident that the decision to opt for conservative kidney management (CKM) is primarily influenced by factors such as older age (85 years), low socioeconomic status, and advanced cancer [5].

Although conservative kidney management (CKM) may be an appropriate option for certain categories of patients, the challenge remains: how can we determine the most suitable treatment approach for each individual? An observational, retrospective study conducted at a single center in the Netherlands aimed to address this question by analyzing 366 patients aged ≥ 70 years with stage 4 or 5 chronic kidney disease (CKD), treated between 2004 and 2016. The objective was to identify predictors of 2-year mortality based on the initial treatment decision, dialysis or conservative kidney management. Predictors included age, eGFR, presence of malignancy, and cardiovascular disease. Separate logistic regression models were developed for each treatment group and internally validated using bootstrapping techniques. Model performance showed moderate discrimination, with optimism-corrected C-statistics ranging from 0.675 to 0.750, and good calibration, indicating reliable prediction of outcomes. [10].

These findings suggest that simple clinical variables (age, eGFR, presence of malignancy, and cardiovascular disease) available at the time of treatment decision can be used to estimate short-term survival and may support shared decision-making in elderly CKD patients [10].

In another observational study conducted on 211 patients with chronic kidney disease (CKD stage 4–5) from a multidisciplinary KF unit at Hospital Universitario de la Princesa in Madrid, Spain, the decision regarding the modality of kidney replacement therapy (KRT) was significantly influenced by clinical, nutritional, and inflammatory parameters. In-center hemodialysis patients had higher Charlson Comorbidity Index (CCI) scores and less favorable biochemical profiles, including lower serum albumin, prealbumin, transferrin, hemoglobin, and eGFR, and higher CRP levels, compared with those on home-based KRT [1].

Taken together, the included studies, originating from diverse settings such as Spain, the United Kingdom, and the Netherlands, suggest that acceptance of conservative kidney management likely varies substantially across regions, influenced by cultural norms, ethnic composition, healthcare system structure, and broader geographic factors. Although none of the included studies were designed to compare cultural or geographic differences, the fact that decision-making around CKM is shaped by patient preferences, social support, and local clinical practice—as described across cohorts from Spain, the UK, and the Netherlands—suggests that substantial global variation in the acceptance of CKM is likely.

**Fig. 2:**
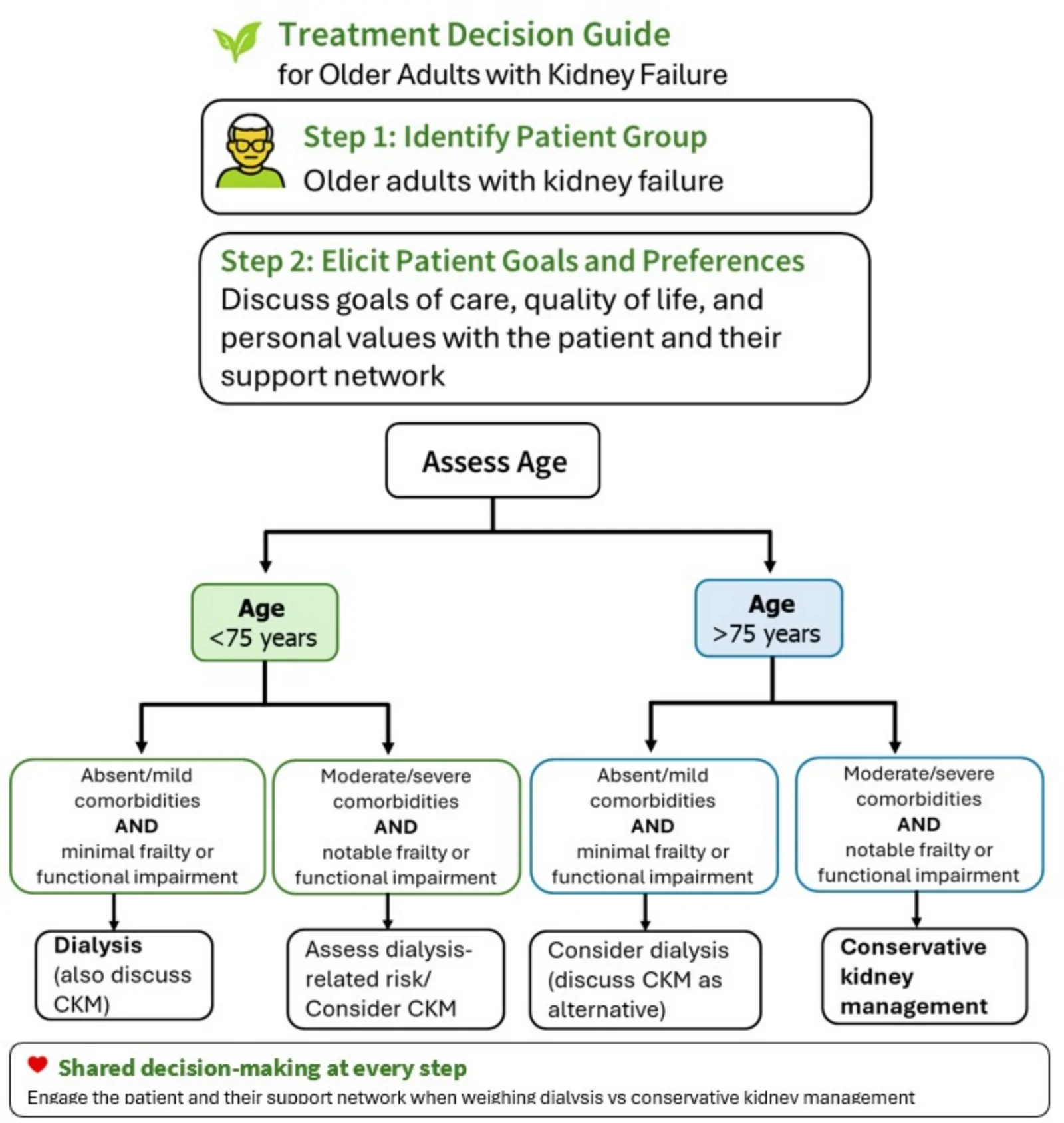
Treatment decision guide for older adults with kidney failure

Taken together, these factors highlight the need for a structured, patient-centered approach that integrates age, frailty, comorbidity burden, and patient preferences when considering dialysis versus conservative kidney management. To facilitate this process in clinical practice, a treatment decision guide is proposed, illustrated in Fig. 2.

This schematic illustrates a stepwise, patient-centered framework for treatment decision-making in older adults with kidney failure. The process begins with identification of the patient group and early elicitation of patient goals, values, and preferences, emphasizing shared decision-making throughout. Subsequent stratification is based on age (< 75 vs. ≥ 75 years), comorbidity burden, and degree of frailty or functional impairment. Patients with absent or mild comorbidities and minimal frailty may be considered for dialysis, while conservative kidney management is favored in those with advanced comorbidities or significant frailty. In intermediate clinical scenarios, individualized assessment of dialysis-related risks and discussion of conservative kidney management as an alternative are recommended. Shared decision-making with the patient and their support network is integral at every stage of the process.

### Withdrawal from dialysis

In incident hemodialysis patients aged 80 years and older, treatment withdrawal represents a frequent and clinically consequential outcome. Evidence from Ko et al. indicates that approximately 10% of these very elderly patients discontinued dialysis, rendering withdrawal the second leading cause of death within this subgroup. The median interval between the last dialysis session and death was 10 days, reflecting the close temporal association of withdrawal with end-of-life. Compared with younger counterparts, patients ≥ 80 years had more than a threefold higher incidence of withdrawal. Determinants of this outcome included advanced age, non-Hispanic white race (in contrast to African American, Hispanic, and Asian patients, who were significantly less likely to withdraw), the presence of dementia, and initiation of hemodialysis with a central venous catheter rather than an arteriovenous fistula [11].

In addition, laboratory and clinical indicators consistent with the malnutrition–inflammation–cachexia syndrome—such as low body mass index, hypoalbuminemia, reduced normalized protein catabolic rate, and elevated leukocyte counts—were strongly associated with dialysis discontinuation, suggesting that frailty and nutritional compromise play central roles in this decision. Notably, even patients who ultimately withdrew had functional and clinical status sufficient to attend thrice-weekly in-center dialysis sessions, underscoring the complexity of decision-making beyond mere treatment feasibility. Geographic variation further influenced outcomes, with patients in certain Midwestern KF networks exhibiting higher odds of withdrawal, while those in New York demonstrated substantially lower rates [12].

Collectively, these findings highlight that dialysis withdrawal in the very elderly is shaped by an interplay of clinical vulnerability, sociodemographic characteristics, and regional practice patterns. The authors also acknowledged several limitations, including the absence of detailed data on symptom burden, functional status, and patient–provider decision-making processes, which may have further informed the context of dialysis withdrawal in this population. [11, 12].

Taken together, these studies consistently demonstrate that while dialysis may extend survival in selected, less comorbid patients, this benefit diminishes in the presence of frailty and multimorbidity. Conservative kidney management, by contrast, offers comparable health-related quality of life and lower hospitalization burden, particularly among the very elderly.

## Recommendations for future study

The body of evidence comparing dialysis with conservative kidney management (CKM) in older adults underscores the complexity of treatment decisions in this population. Survival advantages associated with dialysis shrink or disappear in highly comorbid and frail patients, yet clinicians still lack validated instruments that predict outcomes along the conservative pathway itself.

Observational comparisons show that in patients aged ≥ 75 years, dialysis confers a crude survival advantage overall, but this benefit is substantially attenuated or lost with high comorbidity. These findings argue for nuanced, patient-level prognostication rather than modality-default decisions. Efforts to integrate geriatric assessment—frailty, functional and cognitive status, nutritional markers—into nephrology decision-making are encouraging but incomplete.

Systematic review evidence suggests that HRQoL and symptom outcomes under CKM can approximate those under dialysis, especially in carefully selected patients. However, heterogeneity and selection bias temper confidence, and the absence of CKM-focused prognostic tools remains a central limitation.

### Clinical implications

For clinical practice, these findings emphasize the importance of individualized discussions that integrate geriatric assessment, functional trajectories, and patient preferences when considering dialysis initiation in older adults. Shared decision-making should be anchored not only on life expectancy but also on functional independence, symptom burden, and perceived health-related quality of life.

### Future directions

Future research should prioritize the development and external validation of CKM-specific prognostic tools, derived from cohorts of patients who actively choose conservative kidney management. Such tools should integrate multidimensional domains—frailty, nutritional status, cognitive function, mood, and social context—and report individualized risk over clinically meaningful timeframes. Embedding these models within shared decision-making frameworks could better align care with patient values and reduce uncertainty in prognostication.

The body of evidence comparing dialysis with conservative kidney management (CKM) in older adults underscores how difficult it is to make treatment choices without reliable, CKM-specific prognostic tools. Survival advantages associated with dialysis shrink or disappear in highly comorbid and frail patients, yet clinicians still lack validated instruments that predict outcomes on the conservative pathway itself—the very estimate most patients and families ask for.

Observational comparisons show that in patients ≥ 75 years, dialysis confers a crude survival advantage overall, but this benefit is substantially attenuated or lost with high comorbidity (especially ischemic heart disease), illustrating why one-size-fits-all survival expectations are misleading in geriatric KF care [7]. These findings argue for nuanced, patient-level prognostication rather than modality-default decisions.

Attempts to bring geriatrics into nephrology decision-making (frailty, ADL/IADL, cognitive and nutritional status) are encouraging but incomplete. For example, a Spanish cohort found that age and (pre)frailty predicted a patient’s opting for CKM, yet survival was similar between pathways—supporting the clinical intuition that vulnerability and functional reserve drive both choice and prognosis [8]. However, that work was not designed to yield a CKM-specific prediction tool. Similarly, the Multidimensional Prognostic Index (MPI) correlated strongly with hospitalizations and mortality in mixed CKD populations (CKD stages 3–5 and dialysis), but the model was validated across modalities rather than calibrated to forecast outcomes if CKM is chosen, limiting direct applicability to conservative kidney management counseling. Beyond survival, trajectories that matter to older adults—functional decline, symptom burden, and life impact—are variably captured.

Nursing-home residents initiating dialysis experienced marked and sustained functional decline, illustrating why “living longer” may not equate to “living better” in frail individuals [13]. Yet we lack parallel, prospective tools that forecast functional outcomes under CKM to support preference-concordant choice. Systematic review evidence suggests HRQoL and symptom outcomes on CKM can approximate those on a dialysis pathway in selected elders, but heterogeneity and selection bias temper confidence and again reveal the absence of CKM-focused prognostic instruments [3].

Finally, physician recommendations vary widely and are influenced by beliefs about modality-specific survival across comorbidity profiles—another signal that standardized, CKM-relevant prognostication is missing from routine decisions [5].

#### Implications for future research

Research priorities should shift from dialysis-centric or mixed-modality models to CKM-specific prognostic tools that are (1) developed from cohorts expressly choosing CKM, (2) externally validated, and (3) usable at the point of care. Minimum domains to include:

- Frailty and function: performance-based metrics (gait speed, grip strength) plus ADL/IADL to capture reserve and vulnerability—factors already shown to shape choices and outcomes.
- Comorbidity and disease trajectory: parsimonious comorbidity indices with high-impact conditions (e.g., ischemic heart disease) and kidney function slope (not just level) to reflect dynamic risk.
- Nutrition and sarcopenia: objective markers (albumin trends, weight loss, muscle mass indices) given their ties to hospitalization and mortality.
- Cognition and mood: cognitive impairment and depression, both common and prognostically relevant in older CKD.
- Symptoms and HRQoL: validated patient-reported measures to forecast how patients are likely to live, not only how long.
- Social context: living situation, caregiver availability, and treatment burden tolerance to enhance real-world calibration.

Methodologically, models should be decision-anchored (predicting outcomes conditional on CKM), report individualized absolute risks over clinically meaningful horizons (e.g., 6, 12, 24 months), and include functional and HRQoL endpoints alongside mortality and hospitalization. Embedding these tools within shared decision-making could reduce unwarranted practice variation and align choices with what matters most to older adults considering conservative kidney management.

## Discussion

The evidence synthesized in this review makes clear that the choice between dialysis and conservative kidney management (CKM) in older adults cannot follow a one-size-fits-all approach, as treatment outcomes are shaped far more by individual variation in frailty, functional reserve, comorbidity, and personal goals than by modality alone.

### Summary of main findings

This review demonstrates that treatment decisions for older adults with advanced CKD are highly individualized and influenced more by patient-level factors—age, frailty, functional reserve, cognition, and comorbidity—than by the dialysis versus CKM distinction itself. Across studies, dialysis provides a survival advantage for carefully selected, relatively fit patients, whereas this benefit diminishes substantially in those with significant frailty, cognitive impairment, or functional dependence. Conversely, CKM often yields comparable health-related quality of life, lower treatment burden, and greater alignment with patient-defined priorities such as autonomy, comfort, and time spent at home.

### Interpretation of the evidence

Across cohorts, functional status consistently emerged as a more powerful determinant of outcomes than estimated glomerular filtration rate (eGFR) alone. A steeper decline in eGFR predicted mortality among CKM patients but not among those starting dialysis, underscoring the prognostic value of disease trajectory in the non-dialysis pathway. Frailty, pre-frailty, cognitive impairment, and dependence in activities of daily living frequently guided clinicians and families toward CKM and were strongly associated with higher hospitalization burden.

Prognostic tools—including multivariable mortality calculators and geriatric composites such as the Multidimensional Prognostic Index—provided moderate discrimination and highlighted the central role of biological vulnerability rather than modality choice. Although physical function may initially appear better among patients preparing for dialysis, quality-of-life trajectories between dialysis and CKM tend to converge over time, with CKM often achieving better kidney-disease-specific quality of life due to reduced symptom and treatment burden.

### Strengths and limitations

#### Strengths

A major strength of this review is the comprehensive synthesis of evidence across diverse clinical settings, incorporating studies from multiple countries, health systems, and methodological approaches. By integrating cohorts that vary in age distribution, comorbidity profiles, frailty burden, and care pathways, the review captures the true heterogeneity of the older adult CKD population—an essential consideration when evaluating dialysis versus conservative kidney management (CKM). In addition, the review deliberately combines clinical endpoints (mortality, hospitalization, eGFR trajectory) with functional, cognitive, and patient-centered outcomes (quality of life, treatment burden, independence), offering a more holistic perspective than many prior reviews focused solely on survival. The synthesis also highlights emerging prognostic tools, geriatric assessments, and shared decision-making frameworks, providing clinicians with actionable insights for practice. Finally, by identifying convergent themes across otherwise heterogeneous studies, the review contributes conceptual clarity to an area where clinical uncertainty is common and where real-world decision-making is complex.

#### Limitations

This review is constrained almost entirely to observational evidence; no randomized controlled trials (RCTs) directly comparing dialysis with conservative kidney management in older adults with advanced CKD were identified. The absence of RCTs has several implications:

- No randomized trials. Evidence comes almost entirely from observational studies; causal effects of dialysis vs. CKM cannot be established.
- Bias by indication. Modality choice is entwined with prognosis (age, frailty, cognition, comorbidity), so adjusted comparisons remain vulnerable to residual confounding.
- Inconsistent “time zero.” Studies start follow-up at different points (decision, eGFR threshold, or dialysis start), introducing lead-time/immortal-time biases and hindering synthesis.
- Small, single-center cohorts and variable settings. Limited external validity and potential underpowering for key outcomes.
- Outcome heterogeneity. Mortality windows, hospitalization metrics, functional and PRO measures are not standardized; cross-study pooling is constrained.
- Prognostic tools not CKM-specific. Available models have moderate discrimination and limited external validation for patients choosing CKM.

Although limiting the literature search to PubMed constitutes a methodological limitation, the practical implications of this restriction are likely modest. PubMed indexes the central body of peer-reviewed biomedical research, including the journals in which studies on advanced CKD, dialysis, and conservative kidney management are predominantly published. Given the clinical scope of this narrative review and its aim to synthesize established evidence rather than conduct an exhaustive systematic search, using PubMed alone was considered an acceptable approach for identifying the key relevant studies.

### Conclusion

In older adults with advanced CKD, no single treatment pathway universally optimizes outcomes. Frailty, comorbidity, cognitive and functional reserve, and renal function trajectory outweigh the dialysis-versus-CKM distinction in determining survival, hospitalization, and quality of life. Decision-making should shift from asking “whether to dialyze” toward exploring “how to live best with kidney failure.” A shared, multidisciplinary, and values-based approach—anchored in accurate prognostication and alignment with patient priorities—remains essential to delivering compassionate, individualized, and effective kidney care.

## Data Availability

No datasets were generated or analysed during the current study.
